# SUMOylation is destined for regulatory T cell-related immune dysregulation

**DOI:** 10.1038/s41420-026-02946-x

**Published:** 2026-02-03

**Authors:** Jinxiu Qian, Liuchunyang Yu, Meng Tian, Xiaoyu Li, Xiuyun Bai, Jue Yang, Rongjun Deng, Qiqiong Liu, Aiping Lyu, Cheng Xiao, Yuanyan Liu

**Affiliations:** 1 School of Chinese Materia Medica, Beijing University of Chinese Medicine, Beijing, China; 2 School of Chinese Medicine, Hong Kong Baptist University, Kowloon, Hongkong China; 3 Institute of Clinical Medicine, China-Japan Friendship Hospital, Beijing, China

**Keywords:** Lymphocyte differentiation, Sumoylation, Cell death and immune response, Epigenetics in immune cells, Immune evasion

## Abstract

Regulatory T (Treg) cells perform immunosuppressive functions in rapid response to genetic and environmental stress for maintaining the immune balance, which play a physiological role in preventing autoimmune and inflammatory diseases. Given the highly dynamic and reversible nature of small ubiquitin-like modifier (SUMO) modification, along with the predominant nuclear localization of SUMO paralogs and their associated enzymes, SUMOylation is essential for the flexible regulation of key nuclear processes in Treg cells, such as membraneless organelle formation, genome integrity, and cell cycle progression. Notably, SUMO:SUMO-interacting motif (SIM) interactions facilitate the formation of regulatory complexes that govern cellular processes, and enable crosstalk with other post-translational modifications (PTMs), particularly ubiquitination, phosphorylation, acetylation, and methylation, which are globally harnessed by Treg cells in various contexts to regulate key processes of protein stability, signaling pathways, transcriptional reprogramming, and epigenetic modifications, thereby fine-tuning their immune-regulatory responses. This review explores the multifaceted roles of SUMOylation in Treg cell biology, emphasizing its influence on differentiation, maturation, transcriptional and epigenetic regulation, and metabolic reprogramming. By delineating these pathways, we aim to uncover how dysregulation of SUMOylation may be destined to Treg cells mediated immune disorders, providing a foundation for therapeutic interventions.

## Facts

1. The intrinsic reversibility and nuclear compartmentalization of SUMOylation underpin its irreplaceable regulatory advantage in key nuclear processes.
2. SUMO:SIM interactions establish multivalent scaffolds that enable PTMs crosstalk, allowing Treg cells to flexibly integrate diverse signals across contexts and precisely fine-tune immunoregulatory responses.
3. SUMOylation serves as a gatekeeper in Treg cell biology by regulating key processes of differentiation, maturation, transcriptional and epigenetic regulation, and metabolic reprogramming.
4. This SUMO ‘addiction’ enables Treg cells to rapidly respond to stress, thereby maintaining immune homeostasis or, when dysregulated, driving immune-related diseases.

## Introduction

The immune system relies on a delicate balance between activation and tolerance to effectively defend against pathogens while avoiding autoimmunity. In this regard, regulatory T (Treg) cells are central to sustain immune tolerance and homeostasis by suppressing excessive immune responses [1]. Dysfunctional Treg cells with impaired function have been linked to autoimmune and inflammatory pathologies, including multiple sclerosis, inflammatory bowel disease, systemic lupus erythematosus, rheumatoid arthritis, and type 1 diabetes, as well as chronic infections [2–6]. However, in pathological contexts such as tumor immunity, the same suppressive function of Tregs contributes to immune evasion and cancer progression [7]. Treg cells rapidly differentiate and mature in response to genetic signaling pathways and environmental stimuli of inflammatory sites and tumor microenvironments (TME), which are the two main locations where they exert their immunosuppressive functions with metabolic and oxidative stress [8–10]. Under homeostasis status, Treg cells should maintain lineage stability for stable immunological function, while, to match for the intricate immune response, they are endowed with the properties of dynamic and rapid response, and even flexible [11–13]. The precise molecular mechanisms underlying Treg-mediated immune regulation remain incompletely understood, but emerging evidence highlights the critical role of post-translational modifications (PTMs), particularly SUMOylation (Small Ubiquitin-like Modifier modification), in flexibly orchestrating every aspect of cellular stress to nicely regulate the functional and metabolic adaptive processes in Treg cells [14].

SUMO family molecules share high homology with ubiquitin molecules, as both exhibit highly similar three-dimensional structures characterized by ββαββ folding and a double-glycerol C-terminus [15]. Similar to ubiquitylation, SUMOylation is a reversible and dynamic PTM process [16]. However, unlike ubiquitin, SUMOylation does not directly target proteins for degradation, but instead regulates their protein-protein interactions, membraneless organ formation, subcellular localization, activity, and stability in rapid response to intrinsic and extrinsic stimuli [16]. The SUMO conjugation-related enzymes and sentrin/SUMO-specific proteases (SENPs) are mutually counteractive processes for homeostatic maintenance, which are enriched in nuclear structures of almost all eukaryocyte. Except for covalent binding, SUMO establishes non-covalent attachment with SUMO-interacting motifs (SIMs), in the form of which expands the possibilities of protein-protein interactions, and enables crosstalk between SUMOylation and other modifications as ubiquitination, phosphorylation, acetylation, and methylation. Intriguingly, SUMO processes in Treg cells differentiation and maturation are ubiquitous and necessary, and more importantly, Treg cells should harness the dynamic and versatile properties of SUMOs to rapidly respond to the genetic and environment stress for their signal transduction, cell cycle, transcriptional and epigenetic regulation, and metabolic reprogramming. However, the SUMO process in Treg cells is a double-edged sword, when dysregulated or hijacked by pathological conditions like the TME, may be destined to autoimmune diseases and immunosuppression related tumor progression.

Hence, alterations in protein SUMOylation are essential for Treg cells in both physiological and pathological conditions. In this Review, we illustrate the main SUMO processes, including SUMOylation and deSUMOylation, non-covalent interactions, and the crosstalk between SUMOylation and other PTMs. We then discuss the evidence and mechanisms through which SUMOylation regulates nearly every aspect of Treg cells, including differentiation, maturation, transcriptional and epigenetic regulation, and metabolic reprogramming, as well as how these processes contribute to Treg cell-related immune dysregulation.

## Brief overview of SUMOs

SUMOylation, a conserved eukaryotic PTM, involves SUMO paralogs, associated enzymes, and target proteins, which are particularly enriched in nuclear structures to facilitate the formation of membraneless organelles and regulate key nuclear processes. Protein deSUMOylation is performed by SENPs, which counteract SUMOylation processes to maintain their homeostasis. Except for covalent binding to the lysine (Lys) residues in substrate proteins, SUMOs can non-covalently attach to proteins containing SIMs [17]. The SUMO:SIM interactions bring expansion of modifier bodies, which may enhance the possibility of protein-protein interaction. More importantly, almost all cellular proteins bear SIM, which makes flexible hybrid of SIMs:SUMO chains with other PTMs to conduct corresponding pathway crosstalk. In brief, the reversible and dynamic properties of SUMOs provide platforms for versatile interaction between proteins and extensive crosstalk with other PTMs in response to external and internal stimuli, which hint at global biological processes as genome integrity, cell cycle progression, signal transduction, transcriptional and epigenetic regulation, and cellular metabolism.

### The homoeostasis of SUMOylation and DeSUMOylation in biochemical processes

**Fig. 1:**
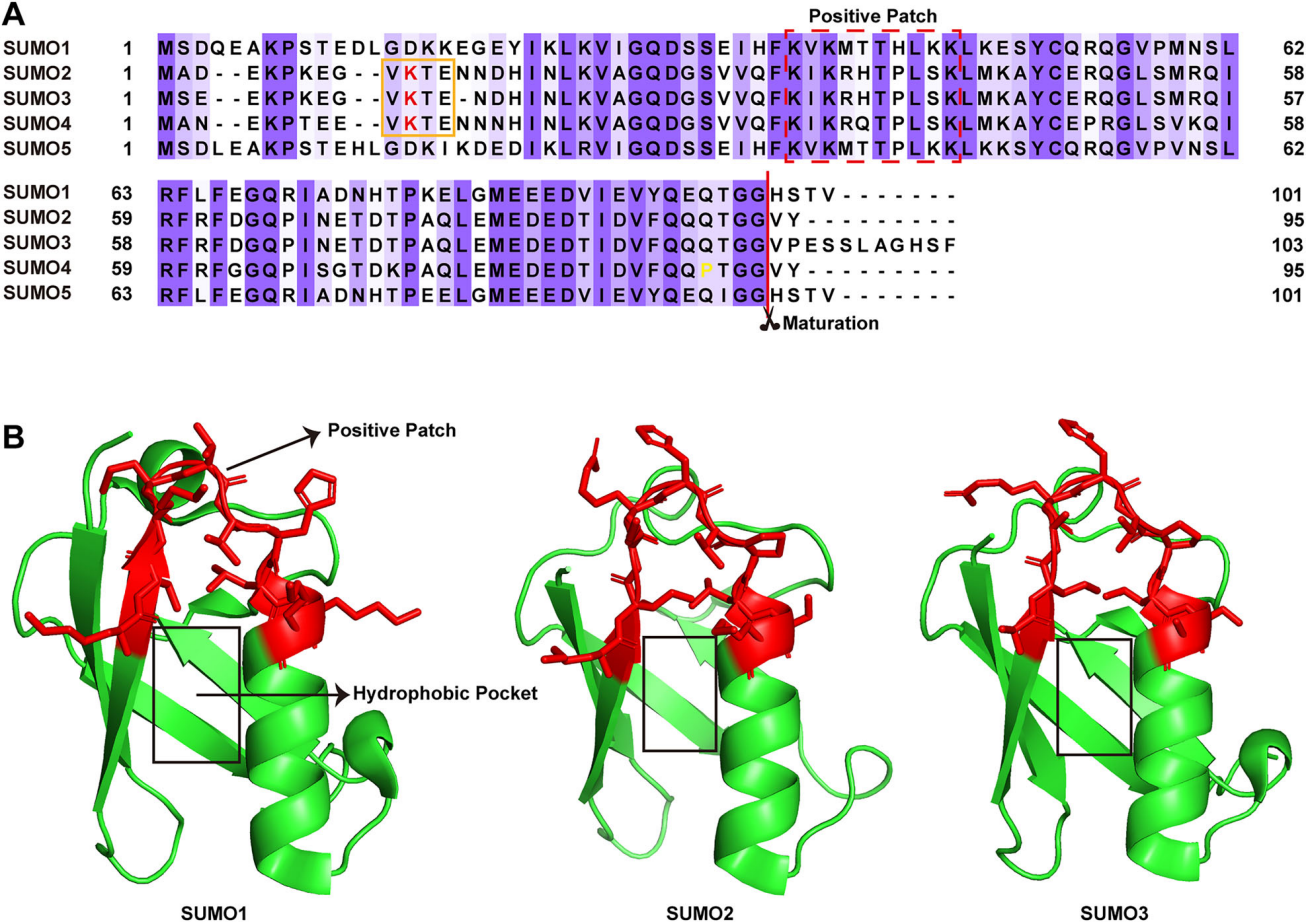
Sequence and structural comparison of SUMO paralogs. **A** Sequence alignment of human SUMO paralogs (SUMO1–5). The consensus SUMOylation motif in SUMO2–4 is boxed in orange, with the lysine (K) acceptor residue in this motif colored red. The positive patch region in SUMO, which binds to the acidic region of SIM, is highlighted by a red dashed box. The cleavage site for the maturation of SUMO paralogs, producing C-terminal diglycine (GG) residues, is also indicated. Additionally, the maturation of SUMO4 is blocked by a unique proline at position 90, which is highlighted in yellow. **B** SIM binding sites on SUMO paralogs. SIMs typically feature a hydrophobic core composed of 3–4 amino acids, which interacts with a conserved hydrophobic pocket (boxed in black) within SUMO paralogs. This core is often flanked by a negatively charged region, which electrostatically interacts with a positively charged patch on the surface of SUMO paralogs (colored red). The structures of SUMO paralogs are shown for SUMO1 (PDB ID 4WJP), SUMO2 (PDB ID 4NPN), and SUMO3 (PDB ID 7ZJU).

**Fig. 2:**
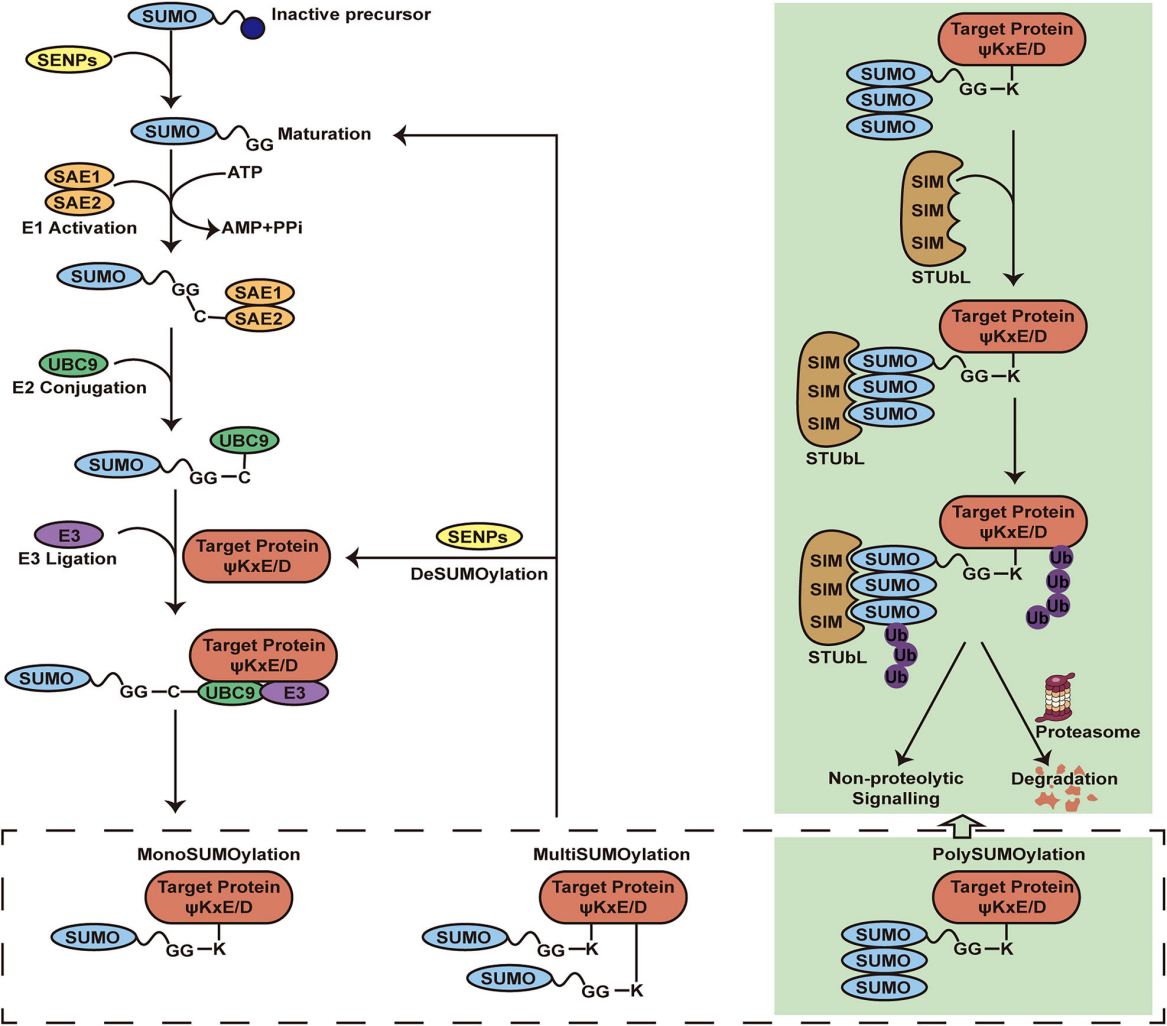
Dynamic SUMOylation cycles in mammalian cells involve sequential enzymatic steps and reversible regulatory mechanisms. SUMO precursors are first processed by SENPs to expose the C-terminal GG motif, enabling their activation via ATP-dependent conjugation to the SAE1/SAE2 E1 heterodimer, forming a SUMO-SAE2 thioester intermediate. The activated SUMO is then transferred to UBC9 E2 conjugase, which covalently attaches SUMO to target protein lysine (K) residues via isopeptide bonds, either independently or with assistance from SUMO E3 ligases that enhance specificity and efficiency. This process results in mono-, multi-, or polySUMOylation of target proteins, which can be dynamically reversed by SENP isopeptidases. Notably, polySUMOylated proteins recruit SUMO-targeted ubiquitin ligases (STUbLs) via SIM domain-mediated recognition of SUMO chains, leading to ubiquitination by STUbL RING domains and subsequent proteasomal degradation or non-proteolytic signaling outcomes.

In mammals, the identified SUMO subtypes include SUMO1–5 (Fig. 1). However, the most commonly expressed subtypes are SUMO1, SUMO2, and SUMO3. In line with this, SUMO1–3 are ubiquitously expressed across most tissues, while SUMO4–5 exhibit tissue-specific expression patterns, with their specific mechanism of action remain unclear [18, 19]. Notably, SUMO2 and SUMO3 exhibit approximately 95% sequence identity and are frequently collectively termed SUMO2/3 [20, 21] (Fig. 1A). In addition, SUMO can modify substrates by attaching to one Lys (monoSUMOylation), multiple Lys (multiSUMOylation), or forming SUMO chains (polySUMOylation) [22] (Fig. 2). Among them, SUMO1 usually form a monomer, whereas SUMO2/3 can form poly-SUMO chains due to their SUMO-binding conserved sequence [22, 23]. Furthermore, SUMO paralogs also show distinct intracellular distribution. SUMO1 primarily localizes to the nuclear envelope and nucleolus, whereas SUMO2/3 are mainly localized to centromeres and condensed chromosomes [24, 25]. Consistent with this, SUMO1 and SUMO2/3 have a certain substrate preference. Interestingly, SUMO1 mainly modifies substrate proteins in normal states [26]. By contrast, SUMO2/3 are predominantly conjugated to substrates under stress conditions [27], which plays a significant role in the adaptation of function and metabolism in Treg cells.

The reversible attachment of SUMO is controlled by an enzyme cascade similar to ubiquitination [16]. However, the SUMO system involves far fewer enzymes than the ubiquitin pathway despite targeting a large number of substrates as well [28]. Before protein SUMOylation, SENPs hydrolyze the C-terminus of SUMO paralogs, exposing their diglycine (GG) motifs for maturation [29, 30]. Conversely, a unique proline at residue 90 prevents SUMO4 from being processed by SENPs, thereby blocking its maturation and rendering it non-conjugatable [31]. After maturation, the SUMO-activating enzyme (E1) [32], composed of the SAE1-SAE2 heterodimer, hydrolyzes ATP to covalently link SUMO to an active-site cysteine. The activated SUMO is then transferred via transesterification to the active site of the SUMO-conjugating enzyme (E2) UBC9, which creates a high-energy thioester bond [33]. The E2 frequently associates with a SUMO ligase (E3) and subsequently catalyzes the conjugation of SUMO to the substrate [34]. SUMO conjugation ultimately forms a reversible isopeptide bond between the C-terminus of SUMO and the Lys residue on the target protein, which can be swiftly reversed by SENPs with isopeptidase activity, enabling SUMO to be recycled for repeated modifications [35, 36]. It is curious to note that protein SUMOylation generally requires a conserved motif with the consensus sequence ψKxE/D, where ψ is a hydrophobic amino acid, K is the target Lys, x is any amino acid, and E/D represent glutamic or aspartic acid [37, 38] (Fig. 2).

Taken together, the enzymes E1, E2, and E3 collectively facilitate SUMOylation, a reversible and dynamic PTM that can be reversed by deSUMOylation through the action of SENPs. The dissimilar subnuclear localization and substrate preference of SUMO paralogs, E3 ligases, and SENP family members suggest that distinct family members have non-redundant roles in coordinating various biological processes. Furthermore, SUMOylation is primarily associated with nuclear functions, including genome integrity, cell cycle progression, signal transduction, transcriptional regulation, epigenetic modification, cellular metabolism, and various cellular stress responses; however, numerous extranuclear substrates have also been reported [39]. Although the SUMO enzymes are less abundant compared to their counterparts in the ubiquitin pathway, there are surprisingly large number of SUMO substrates. SUMO paralogs are covalently bound to thousands of cellular proteins, especially in the nucleus, affecting their activity, stability, localization, and interactions with other proteins. Since SUMOylation is both reversible and highly dynamic, it plays a critical role in enabling Treg cells to rapidly respond to internal and external stimuli, including processes like differentiation, maturation, proliferation, transcriptional and epigenetic regulation, as well as metabolic reprogramming. The dysregulation of SUMO-based homeostasis can directly promote Treg cells mediated immune escape or autoimmune deficiency and is commonly linked with failure of immune response leading to severe tumorigenesis and/or inflammatory disease.

### Non-covalent interactions mediated by SIM

SUMO not only covalently attaches to Lys residues of substrates, but also interacts non-covalently with partner proteins through SIMs [17]. The non-covalent interactions between SUMO and SIM are widespread in SUMO-mediated cellular signaling pathways [17], which transmit intracellular signals by expanding SUMO:SIM interactions to regulate numerous cell functions [40]. Interestingly, SUMO paralogs exhibit distinct specificity in SUMO:SIM interactions, suggesting that they may be involved in distinct biological processes [41].

Generally speaking, the binding affinities of SIMs for SUMOs are weaker than those of covalent bonds but can be strengthened through the simultaneous interaction of multiple SIMs with SUMO chains [40, 42]. The canonical SIM core consists of 3–4 consecutive hydrophobic amino acids, typically surrounded by negatively charged residues, which electrostatically interact with a positive patch on the SUMO surface to enhance SUMO affinity [17, 43, 44] (Fig. 1B). SIMs commonly reside in loops between protein secondary structure elements, and assume extended β-folded conformations upon binding to SUMO [17, 45]. Intriguingly, there is a significant overlap between SUMO targets and SIM-containing partner proteins, which suggests a functional link between covalent SUMO modification and non-covalent interactions [41]. Moreover, many SUMO-related enzymes contain SIMs, which assist in various SUMO processes. For instance, SENP6 and SENP7 both possess multiple SIM-containing domains that preferentially recognize poly-SUMO chains, explaining their predominant role in deSUMOylating substrates modified by polymeric SUMO2/3 [22]. Similarly, enzymes in the SUMO conjugation pathway also contain SIMs, particularly in E3 ligases, where they contribute to the formation of SUMO chains [17].

Emerging evidence indicates that SUMOylation often targets protein clusters, rather than individual proteins, via multiple SUMO:SIM interactions, such as in the formation of PML-NBs [28, 46]. In addition, SIM not only participates in the regulation of SUMOylation but also significantly contributes to the crosstalk of SUMOylation with other PTMs. Collectively, the presence of SIMs enhances the versatile of SUMOylation, influencing protein function in various ways, such as by increasing affinity for SUMO, modulating protein interactions, and affecting crosstalk between different PTMs. This versatility is crucial for Treg cells to perform their immunoregulatory functions in various contexts, such as recruiting transcriptional co-repressors to suppress genes related to effector T cells (as discussed in section "The Roles of SUMOs in pro-Inflammatory Gene Expression and Epigenetic Regulation on Treg Cells").

### The crosstalk between SUMOylation and other PTMs

Given the highly dynamic and reversible nature of SUMOylation, especially the SUMO:SIM association brings versatile properties for additional interactions with other PTMs through the binding of multiple SIMs to SUMOs and pathway crosstalk, which can be globally harnessed by Treg cells in various contexts to regulate key cellular processes and subsequently fine-tune their immune-regulatory responses.

#### Ubiquitination

Ubiquitination is a common PTM process mainly affording to proteins degradation [47, 48]. As the counterpart, SUMOylation shares the similar enzymatic mechanism with ubiquitination, but the involved enzymes are entirely distinct. Both SUMOylation and ubiquitination occur on Lys residues in substrates and can exhibit either competitive or non-competitive inhibition [49, 50]. In some cases, ubiquitin can also be modified by SUMO, although the biological functions are currently unclear [51]. Similarly, SUMOylation can compete with other ubiquitin-like modifications (e.g., ISGylation and NEDDylation) for binding sites, thereby changing the function of substrate proteins [52, 53]. For example, the Lys21 residue in Inhibitor of kappa B (IκB), a site commonly targeted for both ubiquitination and SUMOylation, undergoes degradation through the proteasome pathway upon ubiquitination, whereas SUMO1 modification maintains IκB stability [54]. Besides, polySUMOylation has different consequences on target proteins by subsequent recruitment of STUbLs [17] (Fig. 2).

#### Phosphorylation

Phosphorylation is likely the most common PTM and has consequently been extensively studied [55, 56]. Additionally, phosphorylation is essential for signal transduction, with extensive and complex crosstalk with SUMOylation further regulating these signaling pathways. Investigations into phosphorylation-dependent SUMOylation have revealed an evolutionarily conserved motif comprising a SUMO-binding site and a nearby proline-directed phosphorylation site [57]. Notably, most proteins featuring this motif act as transcriptional regulators, suggesting a pivotal role in gene expression [57]. Furthermore, approximately 9% of the identified SUMOylation sites occur in close proximity to phosphorylation sites, with many shown to rely entirely on preceding phosphorylation events [58]. One reason is that phosphorylation near the hydrophobic core of certain SIMs enhances both specificity and affinity by increasing the negative charge of the SIMs [17, 59], as observed in the transcriptional coregulator Daxx [60] and the DNA repair protein RAP80 [59]. Conversely, evidence for phosphorylation-obstructed SUMOylation was also found. For example, phosphorylation of the AIB1 protein by the MAPK pathway was found to inhibit its SUMO modification [61]. Of particular interest is the observation that SUMOylation and phosphorylation reciprocally modulate the enzymes involved in their processes. Protein kinases show significant enrichment in SUMOylation substrates, thereby promoting the phosphorylation process [62, 63]. In turn, phosphorylation enhances UBC9 activity, leading to increased levels of SUMOylated substrates, thereby regulating protein function to meet specific needs [64, 65].

#### Acetylation

Acetylation, a widespread PTM primarily observed on histone proteins to control transcription, also modulates non-histone proteins involved in essential cellular functions such as metabolism and signaling [66–69]. Histone acetylation enhances gene activation by loosening chromatin structure and creating binding sites for activating factors [68], but studies have shown that H4 histone SUMOylation can block this process by preventing acetylation [70]. Similarly, SUMOylation of several transcriptional factors can recruit histone deacetylases (HDACs) to gene promoters, primarily through SIMs, resulting in reduced histone acetylation and subsequent transcriptional repression [71]. Interestingly, the acetylation of Lys37 in SUMO1 and Lys33 in SUMO2 neutralizes their basic charges, preventing them from binding to SIMs in substrate proteins like PML and Daxx, thus regulating the selectivity and dynamics of SUMO:SIM interactions [72]. Additionally, HDACs and histone acetyltransferases can also be modified by SUMO1, affecting their activity and cellular localization [73].

#### Methylation

Chromatin methylation, including both DNA and histone methylation, is crucial for the stable repression of gene expression [74]. SUMOs tightly regulate this process, primarily by modulating the enzymes or other regulators involved.

DNA methylation, an essential epigenetic modification, is mediated by DNA methyltransferases (DNMTs), which transfer a methyl group to DNA, thereby regulating gene expression [74]. SUMOs have complex effects on DNA methylation, precisely regulating it to control gene expression. On one hand, SUMOylation helps recruit DNA methyltransferases [75]. On the other hand, it regulates their function by enhancing or diminishing enzyme activity or promoting SUMO-dependent ubiquitin-mediated degradation [76–79].

Histone methylation regulates gene expression by modifying chromatin structure or recruiting specific protein complexes, while SUMOylation further modulates histone methylation, influencing both gene activation (e.g., H3K4me3) and repression (e.g., H3K27me3, H3K9me3). For example, SUMO1 distribution on promoters is associated with H3K4me3 to promote transcription [80]. Similarly, SUMOs are also enriched at H3K9me3-marked chromatin regions, and depletion of SUMOs results in a global reduction of H3K9 methylation [81]. SUMO acts as a docking site to recruit silencing effectors with SIMs, such as when SUMOylation of the transcription factor Sp3 recruits SetDB1 and HP1 proteins, leading to H3K9me3 deposition and gene silencing [82]. In addition, SUMOylation connects DNA and histone methylation by forming complexes through SUMO:SIM interactions, further promoting gene silencing [83]. Thus, Treg cells can utilize the mechanism by which SUMOs modulate DNA and histone methylation to precisely regulate the expression of Treg-signature genes and the repression of pro-inflammatory genes.

Moreover, RNA methylation, particularly the N⁶-methyladenosine (m⁶A) modification, constitutes an integral component of epigenetic regulation, modulating a myriad of physiological functions and disease mechanisms [84–86]. Methyltransferase-like 3, an RNA m⁶A methyltransferase, undergoes SUMOylation, which regulates its activity under various conditions [87, 88].

## The roles of SUMOs in differentiation and maturation of Treg cells

The differentiation and maturation of Treg cells are strictly controlled by gene expression for cell activation or differentiation and even costimulation with environmental signals, which may help to maintain immune homeostasis. Intriguingly, the critical roles of dynamic and reversible SUMOylation in cell cycle progression may flexibly dominate the differentiation and maturation of Treg cells, responding dynamically to genetic and environmental stimuli. The SUMO:SIM interactions may contribute to phase separation-mediated nuclear processes, such as subnuclear structures formation and subnuclear localization, which are involved in the early thymic development of Treg cells and play significant role in genome integrity, nucleolar formation, chromosome structures, and corresponding functions. Moreover, SUMO-mediated homeostasis is crucial for regulating diverse cellular processes through competition or crosstalk with other PTMs, thereby influencing cascade events in Treg cell signaling, including the T cell receptor (TCR) signaling pathway, IL-2/STAT5 signaling pathway, and TGF-β/SMAD signaling pathway.

### Early thymic development of T cells

The processes of V(D)J recombination, positive selection, and negative selection are three essential steps in the early thymic development of T cells, all of which are strictly regulated by the dynamic and reversible action of SUMOs to generate functional and self-tolerant Treg cells. Dysregulation of SUMOylation/deSUMOylation can disrupt early thymic development, potentially leading to developmental deficiency of Treg cells.

**Fig. 3:**
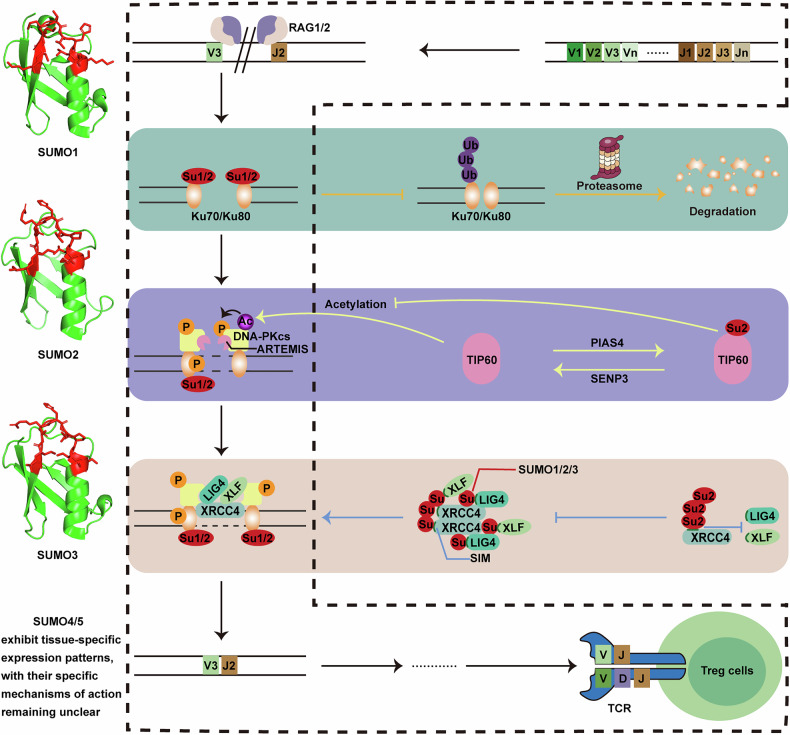
The roles of SUMOs in the mechanism of V(D)J recombination in Treg cells. RAG1/2 trigger V(D)J recombination by recognizing recombination signal sequences and introducing double-strand breaks (DSBs). The repair of these programmed DSBs via the nonhomologous end joining (NHEJ) pathway is tightly regulated by SUMOs, enabling the assembly of diverse antigen receptor genes. Firstly, Ku70/Ku80 binds DNA ends, with SUMOylation (primarily by SUMO1 and SUMO2) stabilizing the complex by preventing ubiquitination and degradation. DNA-PKcs is then recruited and activated via phosphorylation and TIP60-mediated acetylation to process DNA ends. During S-phase, PIAS4-mediated SUMO2 modification of TIP60 reduces its activity, temporally restricting NHEJ. Finally, XRCC4, LIG4, and XLF mediate DNA end ligation, while both non-covalent SUMO:SIM interactions and covalent SUMOylation of XRCC4 orchestrating its localization and interactions in the repair process.

V(D)J recombination assembles and diversifies TCR genes in developing T lymphocytes [89]. In mammalian cells, the nonhomologous end joining (NHEJ) pathway repairs most double-strand breaks (DSBs), including the programmed breaks from V(D)J recombination [90, 91]. SUMOs impact the activity, localization, and stability of essential NHEJ components, such as Ku70/Ku80, DNA-PKcs, and XRCC4 (Fig. 3). In Treg cells, the Ku70/Ku80 heterodimer, essential for NHEJ initiation, is stabilized by SUMOylation, preventing it degradation through ubiquitination [92]. Following the recruitment of DNA-PKcs by Ku70/Ku80, sequential phosphorylation of multiple amino acid clusters on DNA-PKcs triggers DNA end processing by the ARTEMIS nuclease. Moreover, acetyltransferase TIP60 acetylates DNA-PKcs, facilitating its autophosphorylation and activation to promote NHEJ. However, during S-phase, PIAS4 mediates SUMOylation of TIP60, reducing its interaction with DNA-PKcs and limiting its acetylation activity, thereby restricting NHEJ in this phase [93]. This temporal regulation of SUMOylation may ensure that V(D)J recombination takes place at the appropriate stage of lymphocyte development. Once DNA-PKcs is loaded, XRCC4/LIG4 is recruited to facilitate the religation of the broken ends with the assistance of the stimulatory factor XLF [94]. On one hand, both non-covalent SUMO:SIM interactions and covalent SUMOylation of XRCC4 enable its function by localizing it at DNA damage sites [41, 95, 96]. On the other hand, SIMs, based on their distinct locations in XRCC4, can exhibit distinct and even opposing functions, such as promoting or hindering the binding of other key proteins essential for NHEJ-mediated DSB repair, like XLF and LIG4 [41, 97].

After V(D)J recombination, SUMO targets multiple nuclear proteins in response to both internal and external stimuli, thereby ensuring proper maturation and self-tolerance of Treg cells. Accordingly, UBC9-deficient T cells demonstrate defective maturation after positive selection, along with increased apoptosis and impaired proliferation [98]. Furthermore, IKAROS is a hematopoietic cell-specific transcription factor that requires dimerization, and is essential for the successive steps of lymphocyte development and differentiation [99, 100]. Generally, SUMOylation significantly suppresses the activity of IKAROS dimers, disrupting their involvement in both HDAC-dependent and independent transcriptional repression without affecting their nuclear localization [101–104]. Intriguingly, IKAROS deficiency leads to leukemogenesis and lymphomagenesis, typically in conjunction with Notch activation [105]. Indeed, Notch activation is essential for early T cell development but becomes oncogenic after the double positive stage, which is counteracted by IKAROS-mediated repression of the Notch pathway. SUMO conjugation facilitates ligand-induced cleavage of the Notch receptor, a critical step in releasing the Notch intracellular domain (NICD1) from the membrane [106, 107]. Subsequently, NICD1 undergoes SUMOylation, which not only stabilizes NICD1 by reducing its ubiquitination but also enhances its nuclear localization, thereby enabling the transcriptional activation of Notch target genes [106–108]. However, during cell stress, SUMOylated NICD1 reduces transcriptional activity by recruiting HDAC4 to the Notch transcriptional complex [106]. More importantly, RBPJ, a key component of the Notch pathway in the nucleus, assembles the Notch coactivator complex when signaling is active, and inhibits Notch target gene expression when inactive [106]. The SUMOylated non-canonical PRC1.6 creates a conducive environment for recruiting RBPJ to Notch target genes, with the interaction relying on SIMs [109]. On the contrary, the LIM domain protein KyoT2 suppresses the Notch pathway by competitively blocking the RBPJ:NICD1 interaction and recruiting co-repressors like PcG proteins [110]. Moreover, KyoT2 undergoes SUMOylation mediated by PIAS1, potentially weakening its ability to repress transcription in the Notch/RBPJ signaling pathway [110]. Thus, SUMO regulates the Notch signaling pathway in a versatile and complex manner, with IKAROS potentially utilizing this mechanism to modulate the pathway and promote normal T cell development. Thus, SUMOylation-mediated regulation operates in a versatile and complex manner, thereby allowing Treg cells to potentially exploit this mechanism for enhancing early thymic development.

### The roles of SUMOs in nuclear processes of Treg cells

SUMO processes may promote protein-protein interactions and contribute to the formation of membraneless subnuclear structures as nucleolus and chromosomes, which help to maintain genome integrity and assist in the localization and activation of key proteins in Treg cells. Unlike their typical low proliferation ability under normal circumstances, Treg cells exhibit high proliferation in activated tissue environments, particularly within the TME [111]. In this context, SUMOs are crucial for Treg cell proliferation, as its paralogs and enzymes are enriched in nuclear structures, where they globally regulate essential processes such as DNA replication/repair, cell cycle progression, and chromosome segregation.

#### Genome integrity

Treg cells frequently encounter genome integrity challenges, including oxidative stress, hypoxia, and metabolic stress in the TME and inflammatory contexts [112]. DNA damage triggers rapid SUMOylation waves in Treg cells, leading to simultaneous multisite modifications of repair proteins within the same pathway, thereby promoting SUMO:SIM interactions and forming a localized network critical for DDR activation [20, 113–118]. SUMO is recruited to DNA damage sites through the SUMO E3 ligase PIAS1/4 containing a DNA-binding SAP domain, which is essential for the major DNA DSB repair mechanisms of homologous recombination and NHEJ [113]. In a parallel pathway, the SUMO E3 ligase CBX4, a component of PRC1, is recruited to damage sites through the activity of PARP, which is necessary for cellular resistance to ionizing radiation [117].

Chromatin structure regulated by SUMO plays an essential role in assembling protein complexes engaged in DNA repair within Treg cells. For instance, the histone variant H2AX, a crucial component of chromatin, is rapidly phosphorylated in response to DSBs. Phosphorylated H2AX serves as a binding platform for MDC1, facilitating the recruitment of DNA repair factors such as 53BP1 and ubiquitin E3 ligases (e.g., RNF4, RNF8, RNF168, HERC2, BRCA1), which colocalize with SUMO E3 ligases PIAS1/4 at DSB sites [119, 120]. PIAS1/4 are essential not only for the productive association of DNA repair factors with DSBs, but also for the effective formation of ubiquitin adducts at DNA damage sites [113]. For example, MDC1 is SUMOylated by PIAS1/4 after DNA damage, and SUMOylation of MDC1 is required for its degradation and the subsequent clearance of DNA repair factors from DNA damage sites [121]. Thus, appropriate SUMO signalling is involved throughout DNA repair processes, including the recruitment and activation of DDR factors, protein-protein interactions, and ubiquitin-mediated degradation or removal of SUMOylated DDR factors, which are important for maintaining genome integrity in Treg cells [122].

#### Nucleolar formation and function

The nucleolus is the most prominent nuclear body, playing a key role in functions such as ribosome biogenesis, RNA processing, and cell cycle progression [123], all of which are crucial for the differentiation and maturation of Treg cells. As previously mentioned, the nucleolus is enriched with enzymes involved in SUMOylation/deSUMOylation, along with SUMO paralogs. Furthermore, SUMO-deficient cells exhibit significant nucleolar disruption [124]. Additionally, SUMOs are crucial for the formation of other membraneless structures, including PML-NBs and PcG bodies [28, 46]. Given its critical role, it is believed that SUMOs may similarly contribute to the phase separation-mediated nucleolar formation by facilitating protein-protein interactions, although the exact mechanism remains unclear.

Furthermore, maintaining spatial and temporal precision in SUMOylation/deSUMOylation is crucial for proper nucleolar function, particularly in the primary process of ribosome biogenesis. The regulation of ribosome biogenesis by SUMOs is essential for the differentiation and maturation of Treg cells, supporting their complex role in immune regulation by producing the large amounts of protein required. For example, SUMOylated PELP1 promotes MDN1 recruitment to pre-60S particles, and subsequent deSUMOylation releases both MDN1 and PELP1 from pre-60S ribosomes, with this SUMO-regulated coordination of the ordered recruitment and release being essential for pre-60S remodeling [125].

Interestingly, SENP3/5, primarily localized in the nucleolus, play essential roles in ribosome biogenesis, which is vital for meeting the high protein demands of Treg cells in immune regulation. Inactivation of SENP3/5 disrupts distinct steps in the assembly of 40S and 60S ribosomes, triggering both p53-dependent and p53-independent checkpoints, ultimately leading to cell cycle arrest [126]. On one hand, depletion of SENP3/5 leads to excessive SUMO:SIM interactions among pre-ribosomal subunits [126], which can disrupt ribosome assembly critical for Treg cell function. On the other hand, SENP3/5 regulate ribosome biogenesis factors to fulfill the ribosome production requirements of Treg cells. For example, UTP14A, a key regulator of ribosome assembly, is transferred from nucleolus to nucleoplasm in the absence of SENP3/5, and SUMO attachment further hinders its incorporation into pre-ribosomes [126]. Taken together, dynamic and reversible SUMOylation plays a key role in ribosome biogenesis, which is closely related to the cell cycle, and can be utilized by Treg cells to flexibly dominate their differentiation and maturation.

#### Chromosome structure and function

Consistent with the association of SUMO and SUMO pathway enzymes with chromosomes, SUMO deficiency significantly disrupts chromosome integrity and segregation [127, 128], leading to mitotic failure in Treg cells.

Recent research has shed more light on the role of dynamic SUMOylation in regulating specific chromosome structures, including the centromere, kinetochore, and other proteins associated with mitotic chromosomes. A well-characterized example of SUMO regulation of centromere function is its control over constitutive centromere-associated network (CCAN) proteins at kinetochore [129]. The CCAN protein complex consists mainly of certain CENP family members, excluding CENP-A and CENP-E, which are nonetheless closely associated with the function of CCAN. SENP6-mediated deSUMOylation is crucial for the proper localization of CENP family members at centromeres during mitosis [129–132]. Moreover, the depletion of SENP6 leads to polySUMOylation of CCAN proteins, which likely reduces their accumulation at the kinetochore, possibly due to RNF4-mediated proteasomal degradation [129, 131] or steric hindrance caused by SUMO chains [132]. On the contrary, SUMO2/3-mediated SUMOylation is crucial for the localization of the microtubule motor protein CENP-E at the kinetochore, and SENP2 overexpression impairs this targeting without causing protein degradation [24]. Collectively, improper spatial and temporal control of SUMOylation/deSUMOylation at the kinetochore disrupts mitosis in Treg cells.

Another vital SUMO target involved in centromere function is topoisomerase IIα (TopoIIα), essential for decatenating sister centromeres and preventing anaphase bridges during chromosome segregation. Under replication stress, TopoIIα is recruited to stalled replication forks by SNF2-family DNA translocases, where it undergoes SUMOylation by the E3 ligase ZATT, facilitating the SIM-dependent recruitment of PICH to promote extensive fork reversal and restore replication [133]. RNF4, a SUMO-targeted ubiquitin E3 ligase, mediates the ubiquitination and degradation of SUMOylated TopoIIα at stalled replication forks, tightly regulating the ZATT-TopoIIα-PICH axis to prevent excessive fork reversal or collapse [134]. Then during mitosis, PICH also binds SUMOylated chromosomal proteins, particularly SUMO2/3-modified TopoIIα [135], via its SIMs and uses translocase activity to redistribute or remove these proteins and promote tension-induced DNA stretching, thereby resolving chromosome bridges and ensuring proper segregation [136–138]. Concurrently, the precise phosphorylation of SENP3 suppresses its activity toward chromosomal proteins, including TopoIIα, and this phosphorylation-SUMOylation crosstalk ensures chromosome stability [139]. Additionally, PICH can be SUMOylated by SUMO2/3 on mitotic chromosomes, a process highly dependent on the E3 ligase PIASy, consistent with the modification patterns of other mitotic chromosomal SUMO substrates [140]. SUMOylation significantly reduces the DNA-binding capability of PICH; however, it may facilitate the recruitment of other factors associated with ultrafine anaphase bridges [140, 141]. Thus, Treg cells dynamically and precisely regulate the TopoIIα-PICH pathway throughout the cell cycle by directly controlling SUMOylation/deSUMOylation or modulating the crosstalk between SUMOylation and other PTMs, ensuring genetic stability and proper chromosome segregation.

### The roles of SUMOs in signal transduction of Treg cells

**Fig. 4:**
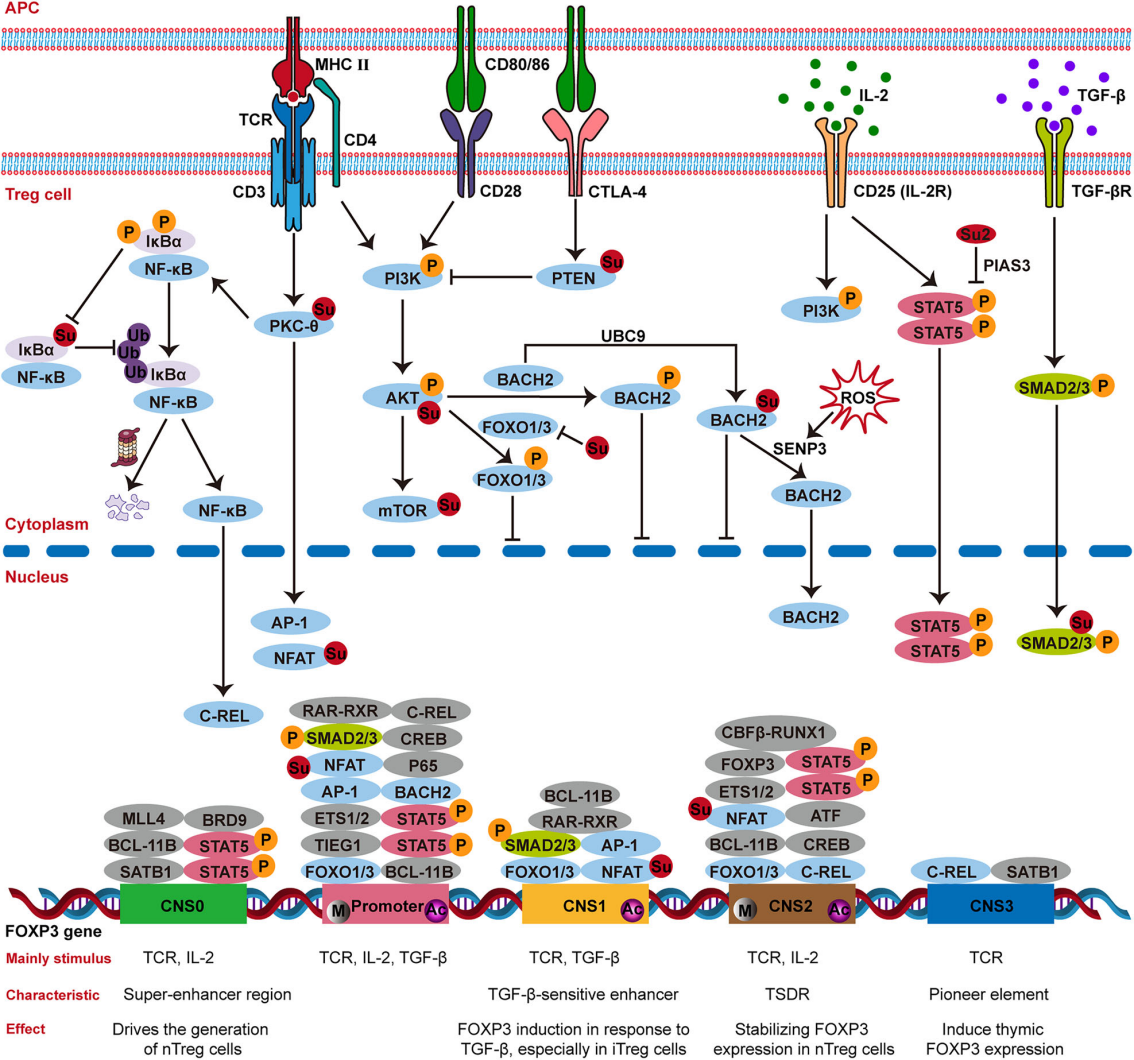
Roles of SUMOs in Treg cell signaling pathways. Treg cell differentiation, activation, and function depend on TCR, IL-2/STAT5, and TGF-β/SMAD pathways that govern FOXP3 expression. SUMOs fine-tune these pathways by modulating protein activity, localization, and interactions. Examples include: SUMOylation enhancing AKT signaling, regulating PTEN to inhibit PI3K-AKT; modulating TCR-related PKC-θ, NFAT, NF-κB, and BACH2; affecting STAT5 in IL-2 signaling; and influencing SMAD2/3 activity in TGF-β/SMAD pathway. P phosphorylation site, Su SUMOylation site, M methylation site, Ac histone acetylation site.

Treg cell development in the thymus and their peripheral immune responses both depend on signal transduction, including key pathways of TCR signaling, IL-2/STAT5 signaling, and TGF-β/SMAD signaling, through which dynamic and stress-responsive SUMO modifications primarily enable Treg cells to rapidly respond to genetic and environmental stimuli for differentiation and maturation (Fig. 4).

#### TCR signaling

The generation of nTreg cells in the thymus requires TCR signals, and mature nTreg cells continuously receive self-reactive TCR signaling in the periphery [142]. Notably, TCR and CD28 signaling activate the PI3K-AKT-mTOR pathway, which has been shown to inhibit Treg cell generation [143]. Phosphatase and tensin-homolog (PTEN), a major negative regulator of PI3K, is constitutively present in naive T cells and primarily downregulates TCR activation, supporting the role of the PI3K-AKT-mTOR pathway in the polarization of CD4 T cell subsets, particularly in maintaining the Th17/Treg balance [144]. PTEN can be SUMOylated by SUMO1 at K266 and K254 [145], which is essential for its translocation to the cell membrane to dephosphorylate PIP3 and inhibit PI3K-AKT signaling [146]. Furthermore, SUMOs induce the AKT kinase activity and the levels of phosphorylated FOXO proteins, primarily under conditions of cellular stress [147]. Thus, Treg cells displayed a consistent defect in AKT phosphorylation at serine 473 and a decrease in phosphorylation of AKT substrates FOXO and S6, which may be partially regulated by SUMOylation [148]. In addition, mTOR, including both mTORC1 and mTORC2 [149], impairs the differentiation of Treg cells [150, 151]. There are multiple SUMOylation sites on mTOR that may affect mTOR signaling through mTORC assembly, localization, and substrate phosphorylation [152].

Protein kinase C-θ (PKC-θ) plays a key role in T cell activation by mediating TCR- and CD28-induced signaling to activate transcription factors [153, 154], thereby promoting cytokine production and T cell proliferation [155]. These transcription factors, such as NF-κB, AP-1, and NFAT, are increasingly recognized as substrates of SUMOylation. For instance, SUMOylation of NFAT regulates its nuclear retention during thymocyte development [98]. Of note, PKC-θ mediates positive feedback in effector T cell activation, while negatively regulating nTreg cell function, although it is necessary for their development. Upon TCR ligation and CD28 engagement, SUMOylation of PKC-θ is essential for its function and the appropriate formation of mature immune synapse [154].

Notably, TGF-β appears to be dispensable for forkhead box protein P3 (FOXP3) induction under conditions of TCR signaling deprivation and PI3K-AKT-mTOR inhibition [143], highlighting that TCR signaling strength are crucial for the differentiation of naive CD4 T cells into induced regulatory T (iTreg) cells [156]. Furthermore, TCR-dependent gene expression landscape heavily relies on SUMOylation, which is why UBC9-deficient Treg cells demonstrate severe defects in TCR-driven homeostatic proliferation, along with impaired activation and compromised immunosuppressive function [157]. Intriguingly, effector T cells require stronger TCR signaling, whereas Treg cells depend on weaker TCR signaling [158]. Additionally, the absence of CD28 results in functional impairment of Treg cells, accompanied by downregulation of CTLA-4, PD-1, and chemokine receptors like CCR6 [159]. Similarly, UBC9-deficient Treg cells also exhibit downregulation of CTLA-4, PD-1, and multiple chemokine receptors [157], suggesting that SUMOs may play key roles in regulating these molecules and maintaining Treg cell function.

#### IL-2/STAT5 signaling

STAT5, a pivotal downstream signaling molecule of IL-2R and a key transcription factor essential for FOXP3 induction, undergoes tyrosine phosphorylation and acetylation upon cytokine activation, leading to dimer formation, nuclear translocation, and transcriptional activation [160]. Phosphorylation of STAT5, regulated by SUMO2-mediated SUMOylation and SENP1-mediated deSUMOylation, is vital for completing its activation-inactivation cycle and returning to the cytoplasm; SENP1 deficiency results in the accumulation of STAT5 in the SUMOylated state, blocking phosphorylation, acetylation, and subsequent signaling [160]. Furthermore, PIAS3 significantly enhances STAT5 SUMOylation through its RING domain activity [160]. In addition, PIAS1, another SUMO E3 ligase, limits the accessibility of the FOXP3 promoter to transcription factors STAT5 through epigenetic modification [75].

#### TGF-β/SMAD signaling

Unlike thymus-derived nTreg cells, iTreg cells differentiate in peripheral lymphoid organs when naive CD4 T cells are activated and exposed to TGF-β [161]. This process induces the expression of FOXP3 [162], which is activated by SMAD2/3, downstream molecules of TGF-β [163, 164]. SUMOylation greatly boosts the transcriptional activity of SMAD2 by promoting its phosphorylation, nuclear translocation, and the formation of the SMAD2-SMAD4 complex [165]. Instead, the SUMOylation of SMAD3 affects its DNA-binding activity and nuclear export [166]. Taken together, SUMOylation strictly regulates TGF-β/SMAD signaling in both time and space, promoting iTreg cell differentiation.

## The roles of SUMOs in lineage stability and epigenetic modifications of Treg cells

FOXP3, BACH2, IKAROS, and IRF4 have been considered as signature genes of Treg cells, sustained expression of which is essential for lineage stability and full immunosuppressive activity. Meanwhile, inhibiting pro-inflammatory gene expression is also crucial for Treg cells to maintain immune tolerance. Of note, the expression of both signature and pro-inflammatory genes can be strictly modulated through SUMO-mediated modifications, in the manner of transcriptional function and epigenetic alteration. Thus, the SUMO-participated gene transcription and epigenetic modification in Treg cells may collectively lead to the loss of immunosuppressive function and, in more severe cases, promote their conversion into pro-inflammatory effector T cells, thereby eliciting autoimmune diseases and influencing immune evasion.

### The roles of SUMOs in signature gene expression and epigenetic regulation of Treg cells

In normal physiological conditions, hypomethylation is essential for Treg cells to maintain their identity and function by stabilizing signature genes (e.g., FOXP3, BACH2, IKAROS, and IRF4) for long-term lineage commitment and full suppressive activity. However, the dysregulation of SUMO-mediated precise gene expression and the crosstalk of SUMOylation with other PTMs, especially methylation and acetylation, enable some conservative regions of Treg cells to be activated and rewritten, then leading to transcriptional and/or epigenetic alterations.

#### Transcriptional function

FOXP3, as the key transcription factor in Treg cells, regulates the expression of multiple genes to orchestrate Treg cell differentiation and function [167]. FOXP3 expression is regulated by TCR signaling via SUMO-mediated pathways that activate transcription factors binding to its promoter or conserved non-coding sequences (CNS). Furthermore, UBC9 deficiency diminished FOXP3 expression during Treg cell expansion [157]. Subsequently, FOXP3 regulates Treg cell functions by activating or repressing specific target genes. Interestingly, FOXP3 has been shown to activate UBC9 gene transcription in MCF7 breast cancer cells [168]. Moreover, the SUMO1 promoter contains potential binding sites for FOXP3 [169]. Therefore, we infer that FOXP3 may promote SUMOylation; however, this hypothesis requires further experimental validation to confirm.

Additionally, other transcription factors regulated by SUMOs play essential roles alongside FOXP3 in directing Treg cell specification and specialized functions [170]. For example, BACH2 promotes Treg cell differentiation by activating signature genes like FOXP3 through enhancer binding, and by repressing TCR-driven activation of pro-inflammatory genes via competition with AP-1 for DNA-binding [171, 172]. TCR- and ROS-mediated SENP3 accumulation triggers BACH2 deSUMOylation, enhancing its nuclear localization and transcriptional activity, which represses genes involved in effector T cell function and stabilizes genes associated with Treg cell identity. [173, 174]. IKAROS cooperates with FOXP3 to repress pro-inflammatory gene transcription [175], with SUMOylation enhancing IKAROS function through dual HDAC-dependent and -independent mechanisms [101–104]. Lastly, SUMOylated IRF4 is crucial for Treg cell differentiation, as it enables IRF4 to function fully in T cells and protects it from proteasome-mediated degradation [176, 177].

#### Epigenetic alteration

SUMOs are key factors in the epigenetic regulation of Treg cells, precisely modulating DNA hypomethylation and histone modifications, such as H3K4me3, through crosstalk with other PTMs to control gene accessibility, thereby influencing transcription and shaping the Treg-specific epigenetic landscape [75, 178].

The DNA hypomethylation pattern is crucial for Treg cells, particularly nTreg cells, as it enhances FOXP3 expression, thereby suppressing inflammation and maintaining immune tolerance. The CpG-rich region of CNS2 within the FOXP3 locus, referred to as the Treg-specific demethylated region, undergoes complete demethylation in nTreg cells [179]. In addition to FOXP3, nTreg cells also exhibit specific hypomethylation patterns in other key genes like CD25, EOS, HELIOS, CTLA-4, and TNFRSF18 [12, 180]. However, UBC9-deficient Treg cells fail to sustain DNA hypomethylation, leading to a significant reduction in the stable expression of activation markers such as FOXP3, CTLA-4, ICOS, CD44, PD-1, and Ki67, especially under proliferative stress. [157]. Similarly, iTreg cells lack this specific hypomethylation pattern, making them unstable and prone to losing FOXP3 expression and other Treg-signature genes, which may lead to their reversion to a conventional effector T cell phenotype [181]. Accordingly, SUMOylation is required to maintain the Treg-specific DNA hypomethylation state, which is essential for the stability of Treg-signature genes, thereby preserving the identity and function of Treg cells.

However, excessive SUMOylation at gene loci can suppress FOXP3 expression, primarily due to its role in transcriptional repression. For example, the SUMO E3 ligase PIAS1 restricts nTreg cell differentiation by sustaining a repressive chromatin state at the FOXP3 locus through recruiting DNA methyltransferases for epigenetic modifications [75]. Notably, the deletion of PIAS1 leads to DNA demethylation, decreased H3K9 methylation, thereby enhancing DNA accessibility. Moreover, SUMOs effect DNA methyltransferases in various ways, including DNMT1, DNMT3A, and DNMT3B. SUMO1-mediated activation of DNMT1 promotes DNA methylation and subsequent downregulation of gene expression [76, 77]. On the contrary, SUMO2/3-mediated SUMOylation of DNMT1 facilitates SUMO-dependent ubiquitination and subsequent degradation [78]. DNMT3A and DNMT3B are also regulated by SUMOs, which primarily diminish their capacity to repress transcription [79, 182]. For example, SUMO1-mediated SUMOylation of DNMT3A disrupts its interaction with HDAC1/2 [182]. Thus, SUMOs are essential for maintaining the hypomethylation of signature genes in Treg cells, particularly nTreg cells, while suppressing gene expression through chromatin modifications, highlighting its critical role in precise spatiotemporal regulation.

Besides DNA hypomethylation, Treg cells also exhibit specific histone modifications, including H3K4me3 [12]. H3K4me3 is a euchromatin marker correlated with a transcriptionally permissive state and is enriched in most Treg-specific hypomethylated regions [12]. Furthermore, the distribution of SUMO1 on promoters is linked to H3K4me3, further promoting transcriptional activation [80]. However, SUMO2/3-mediated protein SUMOylation via E3 ligase CBX4 is crucial for recruiting histone lysine demethylase KDM5B to facilitate the demethylation of H3K4me3 [183, 184]. Strikingly, the SUMOylation level of KDM5B is regulated throughout the cell cycle by targeting the SUMO-targeted ubiquitin ligase RNF4 for proteasomal degradation.

### The roles of SUMOs in pro-inflammatory gene expression and epigenetic regulation on Treg cells

SUMOylation can inhibit pro-inflammatory gene transcription by recruiting SIM-containing co-repressors to SUMOylated transcription factors like NF-κB, STAT, and AP-1. This SUMO:SIM interaction also contributes to heterochromatin formation by recruiting factors involved in repressive histone modifications, such as H3K9me3, thereby ensuring stable pro-inflammatory gene silencing and the epigenetic inheritance of Treg cell identity.

#### Transcriptional regulation

Undoubtedly, inhibiting pro-inflammatory gene expression is crucial for Treg cells to maintain immune tolerance, and this process is intricately regulated by SUMO modification. TAK-981, a potent inhibitor of SUMOylation, disrupts SUMOylation, resulting in increased pro-inflammatory cytokine secretion in lymphocytes, including Treg cells [185, 186]. SUMOylation deficiency also promotes reduced FOXP3 expression, reprogramming intratumoral Treg cells into effector T cells, thereby potentiating antitumor immune response [186, 187]. However, the dysregulation of Treg cell function resulting from SUMO deficiency can also predispose to severe autoimmune diseases.

Generally speaking, Treg cells may utilize SUMO-regulated mechanisms, such as by recruiting SIM-containing co-regulators to SUMOylated transcription factors to inhibit the expression of pro-inflammatory genes, thereby preventing adverse effects associated with immune dysregulation [188, 189]. Specifically, SUMOylation inhibits pro-inflammatory genes (e.g., TNF-α, IFN, and IL-2) by disrupting the activity of related transcription factors like NF-κB, STAT, NFAT, and AP-1 [190]. Notably, Treg cells produce very little IL-2 but are highly efficient at capturing it, supporting their own function while limiting its availability to other effector T cells, thereby enhancing their immunosuppressive activity [167, 191]. SUMOylation of NFATc1 typically represses IL-2 expression by driving NFATc1/C to PML-NBs, where it may interact with histone deacetylases via SIMs, leading to histone deacetylation and transcriptionally inactive chromatin [192, 193]. It is currently known that several SUMO-binding co-repressors are associated with chromatin-modifying complexes, including histone deacetylation and (de)methylation [194]. Strikingly, PML-NBs contain a variety of chromatin-associated proteins, including histones and histone chaperones, which are likely involved in these processes [195]. Similarly, the PcG bodies also consist of a set of proteins responsible for gene silencing and chromatin remodeling [196]. We believe that PML-NBs and PCG contribute to the repression of pro-inflammatory gene expression by promoting the localization and storage of transcriptional co-repressors in Treg cells, a subject deserving further investigation.

SUMOylation also directly regulates the DNA-binding ability of transcription factors, a mechanism that Treg cells can utilize to sustain repression of pro-inflammatory gene expression. For example, classical NF-κB activation involves phosphorylation, polyubiquitination, and subsequent degradation of NF-κB inhibitor (IκBα) [54, 197]. However, the SUMO1 modification of IκBα protects it from degradation, thereby inhibiting NF-κB-dependent transcription [54, 197]. Moreover, SUMOylation attenuates AP-1 activity and thus downregulates the transcription of its target genes, likely due to altered intranuclear distribution, whereas c-Fos (a component of the AP-1 complex) phosphorylation antagonizes its SUMOylation [198, 199].

#### Formation of heterochromatin

Heterochromatin is defined as a chromosomal domain characterized by repressive histone modifications, such as H3K9me2/3 and H3K27me3, along with other associated factors that physically compact the chromatin [200]. SUMOs regulate the formation of heterochromatin through covalent and/or non-covalent attachment to target proteins, which is essential for inhibiting the expression of pro-inflammatory genes in Treg cells.

SUMOylation regulates both H3K9me2/3 and H3K27me3. Here, H3K9me3, catalyzed by histone methyltransferases SETDB1/2 and SUV39H1/2, is used as an example. SUMOylated chromatin regulators (e.g., TRIM28 [201]) or transcription factors (e.g., Sp3 [82]) initiate the establishment of local heterochromatic structures by recruiting chromatin remodeling factor Mi-2, MBT-domain proteins (L3MBTL1 and L3MBTL2) involved in chromatin compaction, and histone methyltransferases (such as SETDB1) that catalyze H3K9me3 [82, 194]. And other cofactors, such as MCAF1, target heterochromatin in a SUMO-dependent manner as well [83]; For more details on additional SUMO-mediated heterochromatin-related cofactors, please refer to other excellent articles on the topic [202]. More importantly, H3K9me3 acts as a binding site for the evolutionarily conserved heterochromatin protein 1 (HP1), a central effector in heterochromatin formation. Specifically, HP1 proteins bind to H3K9me3 through their N-terminal chromodomain, and dimerize via the C-terminal chromoshadow domain, bringing adjacent nucleosomes closer together and promoting chromatin condensation [203]. SUMOs affect the localization of HP1. For example, SUMOylated HP1 targets pericentric heterochromatin, and subsequent deSUMOylation by SENP7 allows HP1 retention at these domains [204]. Mechanistically, SUMOylated HP1 is directed to the pericentromeric heterochromatin domain by specific binding to major forward transcripts [205]. Subsequently, the short module composed of two consecutive HP1-interacting motifs on SENP7 determines HP1 enrichment by restricting its movement at pericentric domains [206]. In addition, SUV39H1 enhances HP1 SUMOylation to target pericentric domains, while its methyltransferase activity increases H3K9me3 levels to create binding sites for HP1 [207, 208]. We believe that the establishment of local repressive chromatin mediated by SUMOylation is also applicable to other nuclear domains and plays essential roles in persistent inhibition of pro-inflammatory gene expression in Treg cells.

Notably, high HDAC levels in heterochromatic regions, with the help of HP1 or other recruitment factors, maintain histone hypoacetylation to inhibit histone turnover and stabilize H3K9me3, thereby ensuring stable gene silencing and epigenetic inheritance [209]. Histones and transcription factors are key substrates of the SUMO conjugation pathway [210, 211], enabling the recruitment of HDAC via SIMs to repress pro-inflammatory genes in Treg cells. Furthermore, HDACs, particularly HDAC1/2, are themselves SUMOylation targets; SUMOylated HDAC recruits other cofactors and facilitate the assembly of repressive chromatin complexes (e.g., NuRD and CoREST) via a network of SUMO:SIM interactions [202, 212].

## The roles of SUMOs in metabolic reprogramming of Treg cells

**Fig. 5:**
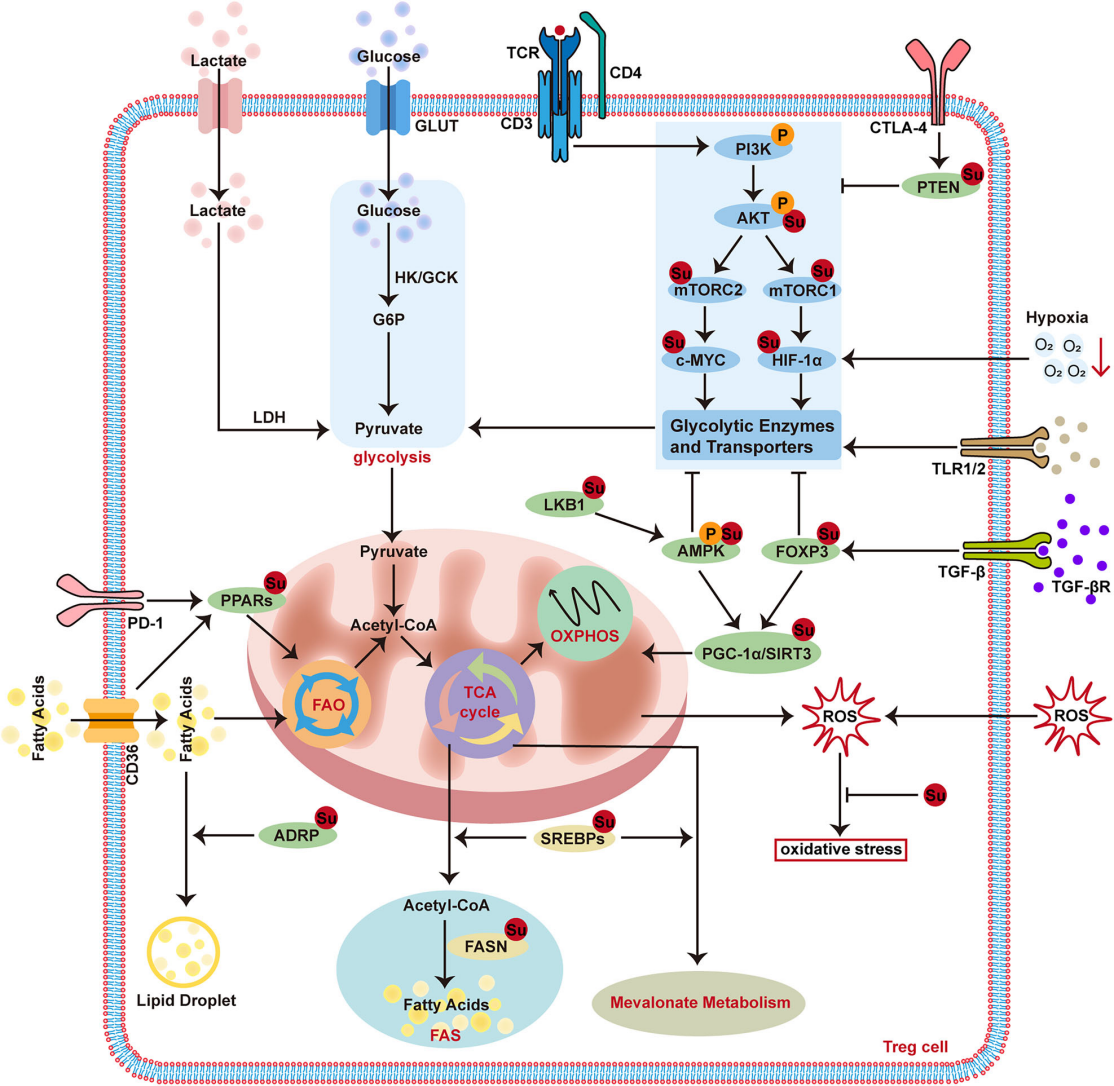
SUMOs govern metabolic reprogramming in Treg cells. SUMO modifications regulate Treg cell metabolic flexibility across glycolysis, mitochondrial metabolism, fatty acid synthesis, and mevalonate metabolism. TCR/CD28-triggered PI3K-AKT-mTOR activation promotes glycolysis for proliferation, yet excessive glycolysis impairs Treg function. SUMOs counteract this by modulating PTEN, AMPK, and FOXP3 to suppress glycolysis and boost mitochondrial oxidative metabolism, enhancing energy efficiency. Additionally, SUMOs regulate mitochondrial function via PGC-1α/SIRT3, influence fatty acid oxidation (FAO) through PPARs, modulate lipid droplet dynamics via ADRP, and control fatty acid synthesis and mevalonate metabolism by targeting SREBPs.

The key feature of Treg cells lies in their ability to flexibly utilize glycolysis and mitochondrial oxidative metabolism, with the latter predominantly comprising fatty acid β-oxidation (FAO) and oxidative phosphorylation (OXPHOS) [213]. Moreover, this metabolic adaptability enables the utilization of alternative metabolites from the environment, thereby supporting their suppressive identity. To some extent, the metabolic flexibility of Treg cells might be attributed to the dynamic and versatile modification of SUMOs to localize and activate key signaling molecules and transcription factors (e.g., HIF-1α, PPARs, and SREBPs), which enable Treg cells to survive and proliferate in harsh environments, outperforming traditional effector T cells that primarily depend on glycolysis to meet metabolic needs. Since the SUMO process is a double-edged sword, in the context of Treg cell-mediated immunological dysregulation, SUMOs can be hijacked by pathological environments and metabolites, not only depriving effector T cells of nutrients, but also supporting the metabolic reprogramming of Treg cells through glycolysis, oxidative metabolism, fatty acid synthesis, and mevalonate metabolism (Fig. 5).

### Glycolysis

Glycolysis reduces the suppressive capacity of Treg cells by promoting IFN-γ production and other effector functions [214], while simultaneously driving their proliferation and metastatic potential [215]. Treg cells may tightly regulate the glycolysis pathway through SUMOs to achieve a balance between proliferation and function in response to both internal and external stimuli. The PI3K-AKT-mTOR pathway, stimulated by TCR/CD28 signaling, is the key driver of glycolysis [213]. SUMOs not only directly regulate the PI3K-AKT-mTOR pathway but also modulate related factors such as FOXP3, PTEN, FOXO1, and HIF-1α, collectively orchestrating Treg cell metabolism and accompanying functional changes.

FOXP3, the metabolic gatekeeper of Treg cells, restricts glycolysis by blocking the c-MYC and mTORC2 pathways [216]. Firstly, SUMOs are essential for both the expression and stability of FOXP3. Then SUMOs also regulate the activity of c-MYC and mTOR. SUMOylation mainly target c-MYC for proteasomal degradation, regulated by SENP1 [217], SENP7, PIAS1, and RNF4 [218]. In contrast, SUMOylation primarily promotes mTOR activity by influencing its intracellular localization and facilitating the assembly of mTOR protein complexes through SUMO:SIM interactions [152]. Additionally, FOXP3 may utilize the transcriptional repression function of SUMOylation to downregulate glycolysis-related gene expression. Together, FOXP3 likely employs SUMOs to regulate the degradation, localization, and assembly of signaling molecules, while also regulating glycolysis-related gene expression, thereby driving the metabolic shift from glycolysis to oxidative metabolism in Treg cells.

PTEN, an upstream inhibitor of PI3K, enhances PD-1 and CTLA-4 expression in Treg cells and relies on SUMOylation to facilitate its nuclear localization. Furthermore, UBC9 deficiency decreased the expression of CTLA-4 and PD-1 in Treg cells [157], suggesting that PTEN may regulate their levels through SUMOylation. Subsequently, ligation of both CTLA-4 and PD-1 in turn activates PTEN to inhibit the upregulation of AKT activity and glycolysis induced by TCR/CD28 signaling [219, 220]. Then SUMOylation of FOXO1 promotes AKT kinase-mediated phosphorylation, thereby preventing its translocation to the nucleus and inhibiting its transcriptional functions [221]. Thus, PTEN regulates the PI3K-AKT-mTOR pathway through SUMOs to drive a metabolic shift that aids Treg cells in adapting to complex environments, while also modulating the expression of activation markers to enhance their immunosuppressive functions.

**Fig. 6:**
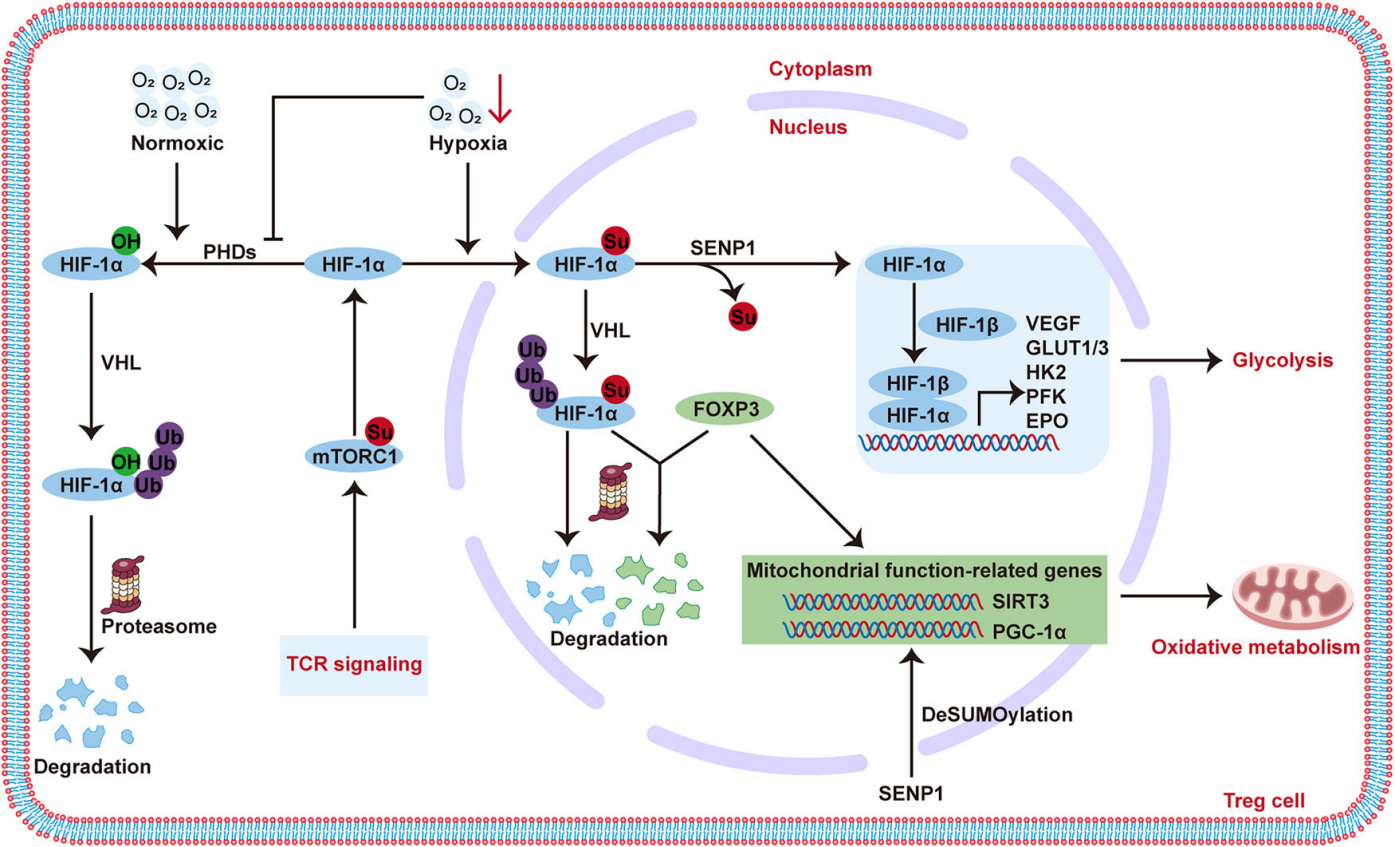
Regulation of HIF-1α function by SUMOs. Under hypoxic conditions, the activity of prolyl hydroxylases (PHDs) is blocked, preventing the hydroxylation of HIF-1α and subsequent ubiquitin-mediated degradation. Simultaneously, hypoxia promotes the nuclear translocation of HIF-1α, a process potentially mediated by its SUMOylation by SUMO1, which also provides an alternative signal for HIF-1α degradation. Moreover, HIF-1α interacts with FOXP3 and facilitates its proteasomal degradation in an oxygen-independent manner. In this case, SENP1 stabilizes HIF-1α through deSUMOylation.

Hypoxia and metabolic dysregulation can impair immune cell development, differentiation, and function, contributing to autoimmune disease pathogenesis [222]. Hypoxia-inducible factor 1 (HIF-1), a downstream molecule of mTORC1, is crucial for cellular adaptation to hypoxia by upregulating the expression of transporters and glycolytic enzymes, including GLUT1/3, lactate dehydrogenase A, hexokinase 2 (HK2) [223], glucokinase (GK), and pyruvate kinase M2 [224], thereby promoting a shift from OXPHOS to glycolysis. Under normal oxygen conditions, cells continuously synthesize and degrade HIFs via the ubiquitin-proteasome pathway [225]. Interestingly, HIF-1α is a well-known substrate for SUMOylation, crucial for cell survival under low oxygen conditions [226] (Fig. 6). Hypoxia promotes the SUMOylation of HIF-1α, which results in its VHL- and proteasome-mediated degradation. In this context, HIF-1α can suppress Treg cell function by enhancing the proteasomal degradation of FOXP3 [227]. SENP1, on the other hand, deconjugates SUMOylated HIF-1α, preventing its degradation under hypoxic conditions, thereby activating HIF-1α-mediated gene expression, such as HK2, GK, PFK, VEGF, EPO, and GLUT1/3. This activation promotes Treg cell migration [228]. Furthermore, SUMOylation also modulates key proteins involved in glycolysis, primarily repressing their associated functions, such as HK2 [229] and GLUT1 [230].

### Oxidative metabolism

In nutrient-deprived, hypoxic environments like the TME, inflammation sites, and the intestine, Treg cells rely on SUMO-mediated metabolic shifts, utilizing fatty acids (via FAO) and lactate to sustain OXPHOS [231, 232]. SUMOs promote metabolic shifts in Treg cells to adapt to environmental changes by regulating the activity, protein interactions, and crosstalk with other PTMs, of key factors such as AMPK, LKB1, PGC-1α, SIRT3, and PPARγ. At the same time, SUMOs protect Treg cells from oxidative stress, thereby supporting their survival and function in high-oxidative stress environments [233].

AMPK, recognized as a key energy sensor, is activated in response to declining energy levels, restoring balance by promoting ATP-generating catabolic pathways, particularly FAO and OXPHOS, which favor oxidative metabolism [234]. Simultaneously, it conserves energy by suppressing ATP-consuming anabolic pathways, including gluconeogenesis, steroidogenesis, and lipogenesis, through mTORC1 inhibition [234, 235]. AMPKα1 is SUMOylated at K118 by the SUMO E3 ligase PIAS4, suppressing its activation and facilitating rapid inactivation of AMPK to restore mTORC1 signaling [236]. However, SENP2 enhances gluconeogenesis by deSUMOylating SUMO2-modified AMPKα, which facilitates its ubiquitin-mediated degradation [237]. Similarly, PIASy specifically conjugates SUMO2 to AMPKβ2, but not AMPKβ1, and enhances AMPK activity by competing with the ubiquitination of the AMPKβ2 subunit [238]. Thus, AMPK is likely primarily modified by SUMO2, with variations in regulatory mechanisms potentially attributed to differences in subunit binding or SUMOylation sites. This flexibility allows Treg cells to adapt their metabolic pathways to environmental conditions by precisely modulating SUMOylation/deSUMOylation at distinct sites.

The AMPK activator LKB1, a key upstream kinase that phosphorylates AMPK and AMPK-related kinases [239–241], activates AMPK to inhibit mTORC1-mediated glycolysis [213]. Upon TCR stimulation, LKB1 protein expression is upregulated in Treg cells but not in effector T cells [242]. This upregulation subsequently leads to the SUMO1 modification of LKB1 at K178, which enables the recognition and activation of AMPK through SIMs [243]. Furthermore, the subcellular localization of LKB1 depends on its binding to STRADα [244, 245]. However, SUMO2 modification of LKB1 at Lys178 inhibits its interaction with STRADα, thereby disrupting its nucleocytoplasmic shuttling due to steric hindrance [246].

Glucose limitation activates AMPK, which in turn engages the SENP1-PGC-1α/SIRT3 signaling axis in mitochondria to improve mitochondrial function and reduce oxidative stress. Specifically, SUMOylation significantly impairs the activation of PGC-1α, whereas SENP1 enhances the transcriptional activity of PGC-1α, which is crucial for mitochondrial gene expression and biogenesis [247]. Furthermore, SIRT3, a key mitochondrial NAD-dependent deacetylase, undergoes SUMOylation in mitochondria, reducing its catalytic activity and increasing mitochondrial protein acetylation, while SENP1-mediated deSUMOylation reactivates SIRT3, promoting FAO and enhancing energy expenditure [248]. Meanwhile, the SENP1-SIRT3 axis decreases the acetylation of mitochondrial SOD2 [249], thereby reducing mitochondrial ROS and safeguarding Treg cells from damage.

In addition to AMPK-related pathways, PPARγ, a lipid-activated transcription factor, plays a critical role in regulating both fatty acid uptake and lipid metabolism in response to lipid ligands [250, 251]. Upon glucose deprivation, Treg cells can proliferate in the presence of lipids, whereas effector T cells cannot [252]. SUMOylated PPARγ is well known to play essential roles in protein stability, subcellular localization, and transcriptional activity [253]. SUMO1-modified PPARγ represses its transcriptional activity, with PIAS1 and PIASxβ functioning as E3 ligases for PPARγ [254, 255]. Overexpression of SENP2 significantly increases the transcription of FAO-related genes, such as CPT1B and ACSL1 [256]. Furthermore, free fatty acids boost SENP2 transcription, which in turn upregulates FAO-related enzymes by deSUMOylating PPARβ and PPARγ [257]. In addition, PD-1 mediates the metabolic adaptation and intratumoral survival of Treg cells [258] by activating the PPARβ pathway [259] and key enzymes in the FAO pathway [220], thereby maintaining mitochondrial lipid metabolism and increasing lipid uptake.

Indeed, to mitigate the toxicity caused by fatty acid accumulation, Treg cells not only enhance mitochondrial FAO but also store fatty acids in lipid droplets (LDs) [260]. Moreover, SUMO1 depletion disrupts the expression of ADRP, a key structural component of LDs, potentially impairing LD formation and function [261].

Mitochondrial oxidative metabolism is a major source of endogenous ROS, while the inflammatory environment and TME are also rich in ROS [251]. These intracellular and extracellular ROS can induce oxidative stress in T cells, impairing their function and potentially explaining why effector T cells avoid oxidative metabolism. Unlike effector T cells, Treg cells effectively counteract ROS by upregulating key molecules such as GSH [262], PDPK1 [263], and GPX4 [264], with this process regulated by SUMOs. For example, UBC9 deficiency disrupts PDPK1 signaling and glycolysis [265]. GPX4 contains three potential SUMOylation sites at Lys74, Lys106, and Lys125 [266]. Among these, SUMOylation at Lys125 may promote its ability to inhibit lipid peroxidation [266, 267].

Furthermore, beyond regulating key molecules that combat oxidative stress, SUMOs also play an important role in sustaining cellular homeostasis under oxidative stress through other mechanisms in Treg cells. Under resting conditions, SENP3 is continuously degrade via the ubiquitin-proteasome pathway [268]. Upon ROS exposure, cysteines in the redox-sensing domain of SENP3 are oxidized, preventing its ubiquitination and triggering its translocation from the nucleolus to the nucleoplasm [268]. In Treg cells, SENP3 rapidly accumulates following TCR and CD28 stimulation in a ROS-dependent manner [173]. It plays an essential role in regulating the stability and function of Treg cells by promoting BACH2 deSUMOylation and preventing BACH2 nuclear export, thereby preserving Treg cell-specific gene signatures while repressing effector T cell transcriptional programs [173]. Accordingly, appropriate levels of ROS are beneficial to the function of Treg cells. Consistent with this, pharmacological studies indicate that reducing ROS levels diminishes Treg cell-mediated immunosuppression and boosts antitumor T cell responses [173, 269]. Therefore, compared to effector T cells, Treg cells have reduced sensitivity to oxidative stress, which aids their survival in the high-oxidative stress environment of the TME [233].

### Fatty acid synthesis and mevalonate metabolism

Fatty acid synthesis and mevalonate metabolism are essential for the proliferation and function of Treg cells. Fatty acid synthesis likely plays a more pivotal role than fatty acid uptake in shaping the lipid pool of Treg cells and promoting their proliferation [270]. SUMOylation safeguards FASN-driven de novo fatty acid synthesis by blocking its proteasomal degradation, thus ensuring lipid metabolic homeostasis [271].

**Fig. 7:**
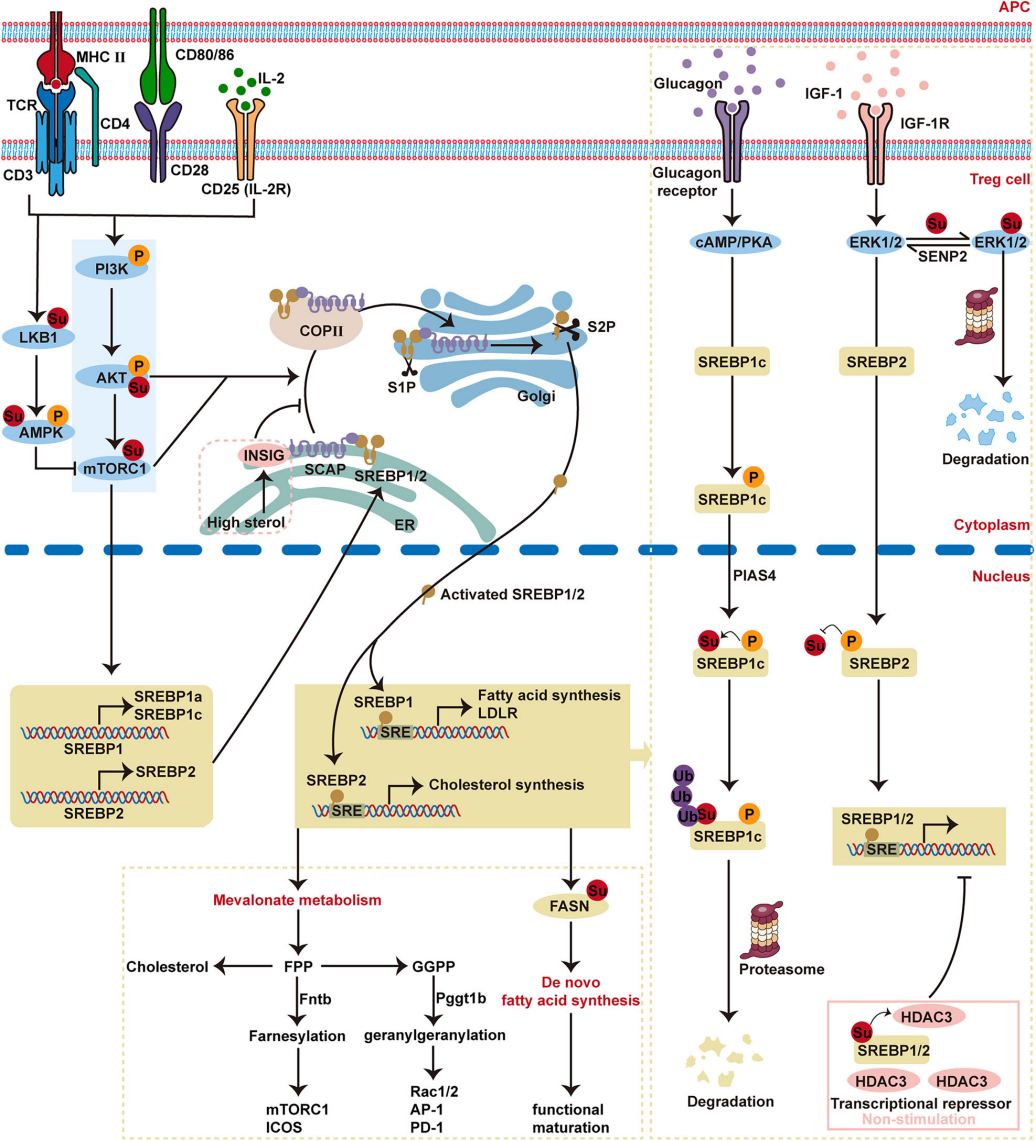
Regulation of SREBP function by SUMO pathways. This figure illustrates the regulation of SREBPs (SREBP1a, SREBP1c, and SREBP2), key transcription factors involved in cholesterol and fatty acid biosynthesis, through SUMOylation and phosphorylation. SUMOylation represses SREBP activity by recruiting co-repressors or inducing ubiquitin-mediated degradation. Conversely, IGF-1 signaling triggers phosphorylation that reduces SREBP2 SUMOylation, thereby enhancing sterol metabolism (Simultaneously, SUMOylation precisely regulates ERK2 SUMOylation, driving its ubiquitin-proteasomal degradation). During fasting, glucagon-activated PKA phosphorylates SREBP1c, increasing its SUMOylation, promoting degradation, and suppressing lipogenesis. These pathways highlight the dynamic interplay of SUMOylation and phosphorylation in regulating lipid metabolism under different physiological conditions.

Strikingly, activation of mevalonate metabolism, primarily regulated by SREBPs, enhances the suppressive function of Treg cells. SREBPs, major transcription factors of lipid homeostasis [272], have upregulated activity in intratumoral Treg cells [273]. Ubiquitination, SUMOylation, and phosphorylation regulate the fate and activity of SREBPs [274, 275] (Fig. 7). In mammals, the SREBP1 and SREBP2 genes encode three isoforms (SREBP1a, SREBP1c, and SREBP2) [272], with SUMOylation inhibiting SREBP-dependent transcription either through transcriptional repression or induced degradation [274–276]. SUMO1 modification negatively regulates SREBPs by recruiting an HDAC3 co-repressor complex [275]. Under specific physiological conditions, IGF-1 activates ERK1/2 to phosphorylate SREBP2, which reduces its SUMOylation and upregulates genes involved in sterol metabolism, such as LDL receptor, squalene synthase, and HMG-CoA synthase [275]. Similarly, SUMOylation of SREBP1c at Lys98 by the SUMO E3 ligase PIAS4 inhibits its activity, thereby reducing hepatic lipogenesis during fasting [276]. During fasting, glucagon from the pancreas activates PKA, which phosphorylates SREBP1c on serine 308, leading to increased SUMOylation and subsequent ubiquitin-mediated degradation of SREBP1c, thereby inhibiting lipogenesis [276]. Simultaneously, signaling molecules involved in the process are precisely regulated by SUMOylation. For example, ERK2 has been reported to undergo SUMOylation, a modification potentially affecting SREBP2 function. Specifically, this SUMOylation on ERK2 further drives its ubiquitin-proteasomal degradation, whereas SENP2 counterbalances this effect by removing the SUMO group from ERK2 [277].

## Conclusion

SUMOylation, a reversible and dynamic PTM, is mediated by E1, E2, and E3 enzymes and is counteracted by SENPs to maintain its homeostasis, and whilst its specific SUMO:SIM interactions provide a versatile protein-protein interaction manner and enable crosstalk with other PTMs. Leveraging all the abovenature, Treg cells utilize SUMOylation to adapt to cellular stress, orchestrating responses that effectively regulate their immunosuppressive functions. As SUMOs and their associated enzymes are enriched in nuclear structures, they can globally orchestrate Treg cell differentiation and maturation by regulating nuclear processes such as cell cycle progression, chromosome segregation, and the subnuclear structures formation and localization, thereby responding dynamically to genetic and environmental stimuli. Remarkably, SUMO:SIM interactions introduced protein regulatory functions and precise spatiotemporal network that enables Treg cells to rapidly adapt and optimize their gene transcription, epigenetic modification and metabolic reprogramming in response to changing demands, might also be harnessed by pathological processes for destined dysregulation. As SUMO ‘addiction’ endows Treg cells with dynamic, rapid, and flexible responses to adapt to complex immune reactions, targeting SUMO processes in Treg cells holds significant potential for therapeutic intervention in autoimmune diseases and cancer, warranting further investigation into its clinical applicability.

## Data Availability

All data generated or analysed during this study are included in this published article.
